# Disability and self-care living strategies among adults living with HIV during the COVID-19 pandemic

**DOI:** 10.1186/s12981-021-00413-4

**Published:** 2021-11-19

**Authors:** Kelly K. O’Brien, Ahmed M. Bayoumi, Soo Chan Carusone, Aileen M. Davis, Rachel Aubry, Lisa Avery, Patricia Solomon, Kristine M. Erlandson, Colm Bergin, Richard Harding, Darren A. Brown, Jaime H. Vera, Steven E. Hanna

**Affiliations:** 1 Department of Physical Therapy, University of Toronto, 500 University Avenue, Room 160, Toronto, Ontario M5G 1V7 Canada; 2 Institute of Health Policy, Management and Evaluation (IHPME), Dalla Lana School of Public Health, University of Toronto, 155 College Street, 4th Floor, Toronto, Ontario M5T 3M7 Canada; 3 Rehabilitation Sciences Institute (RSI), University of Toronto, 500 University Avenue, Room 160, Toronto, Ontario M5G 1V7 Canada; 4 MAP Centre, St. Michael’s Hospital, 30 Bond Street, Toronto, Ontario Canada; 5 Department of Medicine, University of Toronto, Medical Sciences Building, 1 King’s College Circle, Toronto, Ontario Canada; 6 Casey House, 119 Isabella Street, Toronto, Ontario Canada; 7 McMaster Collaborative for Health and Aging, McMaster University, Hamilton, Ontario Canada; 8 University Health Network, 190 Elisabeth Street, Toronto, Ontario Canada; 9 Dalla Lana School of Public Health, University of Toronto, 155 College Street, Room 500, Toronto, Ontario Canada; 10 School of Rehabilitation Science, McMaster University, 1400 Main Street West, Room 403, Hamilton, Ontario Canada; 11 University of Colorado Denver- Anschutz Medical Campus, 13001 East 17th Place, Aurora, CO 80045 USA; 12 The GUIDE Clinic, St. James’s Hospital, Hospital 5, James’s Street, Dublin 8, Ireland; 13 School of Medicine, Trinity College Dublin, 152-160 Pearse Street, Dublin, Ireland; 14 Florence Nightingale Faculty of Nursing Midwifery and Palliative Care, Cicely Saunders Institute, King’s College London, Bessemer Road, London, UK; 15 Therapies Department, Chelsea and Westminster Hospital NHS Foundation Trust, 369 Fulham Road, London, SW10 9NH UK; 16 Department of Global Health and Infection, Brighton and Sussex Medical School, BSMS Teaching Building, University of Sussex, Brighton, UK; 17 Health Research Methods, Evidence and Impact, McMaster University Medical Centre, McMaster University, 1280 Main Street West, Hamilton, Ontario Canada

**Keywords:** HIV/AIDS, COVID-19 pandemic, Web-based survey, Disability

## Abstract

**Background:**

Events associated with the COVID-19 pandemic, such as physical distancing, closure of community services, postponement of health appointments, and loss of employment can lead to social isolation, financial uncertainty, and interruption of antiretroviral adherence, resulting in additional health-related challenges (disability) experienced among adults living with chronic illness such as HIV. ‘Living strategies’ is a concept derived from the perspectives of people living with HIV, defined as behaviors, attitudes and beliefs adopted by people living with HIV to help deal with disability associated with HIV and multi-morbidity. Our aim was to describe disability among adults living with HIV and self-care living strategies used during the COVID-19 pandemic.

**Methods:**

Adults living with HIV in Toronto, Ontario, Canada, including some with pre-pandemic HIV Disability Questionnaire (HDQ) data, completed a cross-sectional web-based survey between June–August 2020. The survey included the HDQ and questions about self-care living strategy use during the pandemic. We compared disability (HDQ) scores prior to versus during the pandemic using paired *t*-tests. We reported the proportion of participants who engaged in various living strategies at least ‘a few times a week’ or ‘everyday’ during the pandemic.

**Results:**

Of the 63 respondents, 84% were men, median age 57 years, and 62% lived alone. During the pandemic the greatest disability severity was in the uncertainty [median 30; Interquartile range (IQR): 16, 43] and mental-emotional (25; IQR: 14, 41) domains. Among the 51 participants with pre-pandemic data, HDQ severity scores were significantly greater (worse) during the pandemic (vs prior) in all domains. Greatest change from prior to during the pandemic was in the mental-emotional domain for presence (17.7; p < 0.001), severity (11.4; p < 0.001), and episodic nature (9.3; p < 0.05) of disability. Most participants (> 60%) reported engaging a ‘few times a week’ or ‘everyday’ in self-care strategies associated with maintaining sense of control and adopting positive attitudes and beliefs.

**Conclusions:**

People living with HIV reported high levels of uncertainty and mental-emotional health challenges during the pandemic. Disability increased across all HDQ dimensions, with the greatest worsening in the mental-emotional health domain. Results provide an understanding of disability and self-care strategy use during the COVID-19 pandemic.

**Supplementary Information:**

The online version contains supplementary material available at 10.1186/s12981-021-00413-4.

## Introduction

Adults living with HIV can experience complex physical, mental and social health-related consequences of multimorbidity, known as disability [1–7]. Events associated with the COVID-19 pandemic, such as physical distancing, closure of community services, postponement of health appointments, and loss of employment can lead to social isolation, financial uncertainty, and interruption of antiretroviral adherence, further adding to disability for adults living with chronic illness such as HIV [8–10]. Disability, in combination with health inequities, stigma, interrupted access to services, substance use, and food or home insecurity during the pandemic, can pose barriers to adults with HIV remaining engaged in care [11–14]. During a pandemic, these stressors may exacerbate disability, placing individuals with HIV at risk of poorer adherence to antiretroviral therapy [15] and poor physical and mental health, associated with poorer retention in care [10, 16]. However, the extent to which the pandemic may affect the severity and presence of disability among adults with HIV is unknown.

Due to measures implemented to slow the transmission of COVID-19, adults living with HIV need to employ independent self-care living strategies in order to deal with uncertainty and the physical, social and mental health challenges of the pandemic [9, 13]. Living strategies is a term that may be considered analogous to coping in the context of HIV. The concept of ‘living strategies’ was derived from the perspective of people living with HIV as a component of the Episodic Disability Framework, defined as behaviors, attitudes and beliefs adopted by people living with HIV to help deal with disability associated with HIV and multi-morbidity [7]. Examples of living strategies include: seeking social interaction with others, maintaining a sense of control over life, blocking the event (i.e., the COVID-19 pandemic) out of the mind, and maintaining positive attitudes and beliefs [7]. While most strategies have positive influences on health, some strategies may have negative health consequences, such as isolation (associated with physical distancing measures), or engaging in substance use. Understanding how these living strategies are used among adults living with HIV during this time can help to identify ways in which individuals and health care providers can promote timely and appropriate self-management approaches for enhancing health and well-being during a pandemic or other major events [17, 18].

Our aim was to describe disability experienced by adults with HIV and self-care living strategies used during the COVID-19 pandemic. Our primary objective was to describe the dimensions of disability experienced among adults with HIV during the pandemic and identify change in disability compared with prior to the pandemic. Our secondary objectives were to describe self-care living strategies used; and examine associations between disability and the frequency of living strategies use; and to identify changes in mental health and mastery compared with prior to the pandemic.

## Methods

### Study design

We conducted a cross-sectional web-based survey using EQUATOR guidelines for web-based surveys [19, 20]. This research was approved by the HIV/AIDS Research Ethics Board at the University of Toronto (Protocol #32910).

### Setting

In response to the World Health Organization’s declared outbreak of the novel coronavirus disease (COVID-19) the province of Ontario, Canada declared a state of emergency on March 17, 2020. To slow the transmission of SARS-CoV-2, the City of Toronto closed all shared, public or private recreational amenities and non-essential businesses, including community service organizations and health and fitness centres, and issued a stay-at-home order.

### Participants

Individuals were eligible if they self-identified as an adult ≥ 18 years living with HIV living in Toronto, Canada.

### Recruitment

We emailed adults living with HIV who participated in a community-based exercise (CBE) intervention study [21] and agreed to be contacted about future research. Using a modified Dillman Tailored Design Method, we emailed potential participants an initial invitation and link to electronically administer, self-reported questionnaires using Qualtrics [22, 23]. The email outlined the study purpose, eligibility, and involvement. Interested individuals opened a personalised Qualtrics link, consented to participate by clicking yes to ‘I agree to participate in this research study’, and were the guided to the survey. We followed up with reminder emails at one, two and four weeks after the initial invitation. Participants received a $30 (Canadian dollar) electronic gift card for completing the survey. To increase our sample size, we asked participants to share details of the study with others living with HIV, who were asked to contact the research coordinator to review eligibility prior to enrolling in the study and receiving a personalized link to the survey.

## Data collection

The survey included questionnaires capturing disability, self-care living strategies use, contextual factors (mental health, mastery), and COVID-19, health and demographic characteristics of participants. For participants recruited from the exercise study, we used HDQ data collected during that study (prior to the pandemic) to assess change in disability.

### Disability

We administered the HIV Disability Questionnaire (HDQ), to capture the presence, severity and episodic nature of disability experienced by adults aging with HIV across six domains including challenges with physical, mental-emotional, and cognitive health, day-to-day activities, social inclusion and uncertainty or worrying about the future [24, 25]. The HDQ has demonstrated validity, reliability and sensibility for use with adults living with HIV [26–30]. The HDQ captures uncertainty and the episodic nature of disability, making it an ideal tool in which to measure disability during the COVID-19 pandemic*.*

### Self-care living strategies

We administered a Living Strategies Questionnaire, adapted from categories in the Episodic Disability Framework and previously administered with adults with HIV in Canada [31]. The 51-item questionnaire captured the frequency of living strategy use including: maintaining a sense of control in the context of uncertainty (lifestyle—physical activity, sleep health, nutrition, daily routine, re-establishing purpose, maintaining life balance, planning for and anticipating the future, and paying attention to health) (26 items); attitudes and beliefs (outlook on life, faith and spirituality) (eight items); blocking the COVID-19 pandemic out of the mind (seven items), and a section on seeking social interactions with others (10 items). For items 1–41, participants were asked how often they used each living strategy in the past month ranging from 1 “None of the time (e.g., not at all)” to 4 “All of the time (e.g., every day)”. For the seeking social interaction items (Item 42–51), participants were asked how often they used each social strategy in the past month ranging from 1 “None of the time (e.g., not at all)” to 5 “All of the time (e.g., every day)” or not applicable. For each, we asked whether the frequency of strategy use changed since the onset of the COVID-19 pandemic: increased (more frequent), decreased (less frequent) or no change.

### Personal characteristics and COVID-19 factors

We administered a demographic and COVID-19 questionnaire to capture personal characteristics (age, sex, gender, living status, income, employment status), HIV-related information (viral load, years since diagnosis, concurrent health conditions, self-reported health), and impact of the COVID-19 pandemic on access to health care services, supports, experiences and lifestyle changes.

### Secondary measures

#### Mental health

The Patient Health Questionnaire (PHQ8) is an 8-item measure of depression severity [32] and possesses construct validity and test–retest reliability in adults living with HIV [33, 34].

#### Mastery

The Pearlin Mastery Scale includes seven items that assess sense of personal control over life forces or outcomes [35]. Each item in the scale is measured using a Likert scale with four response categories, and higher summary scores indicate greater levels of mastery.

### Analysis

We downloaded responses from Qualtrics for analysis using R (analytic analyses) [36]. We calculated the view, participation and completion rates of survey responses [19]. We calculated medians (IQR) and frequencies (%) to describe demographic characteristics of the sample. We conducted a cross sectional analysis with all participants who completed the web-based survey during the pandemic, and longitudinal analysis (prior to versus during the pandemic) among participants with pre-pandemic disability (HDQ), mental health (PHQ8) and mastery (mastery scale) data.

#### Disability

We calculated medians and interquartile ranges (IQR) for HDQ presence, severity and episodic domain scores from all survey respondents. For participants recruited from the exercise study, we used paired t-tests to determine if their disability changed prior to versus during the pandemic. We used each participant’s median pre-pandemic HDQ score across all possible 12-time points in our previously 22 month exercise study [21] as the pre-pandemic score, and the current HDQ score from the web-based survey as the during pandemic score.

#### Self-care living strategies use

We reported the frequency (%) of participants who engaged in each strategy ‘a few times a week’ or ‘everyday’ and change in strategy use (increase/decrease/no change). We conceptualized 37 of the items as having positive influences on health, 8 having negative influences on health, and 6 as having either a positive or a negative influence on health, dependent on the individual and context [31].

#### Secondary (exploratory) analyses

We calculated median (IQR) scores for the PHQ8 (score range: 0–24) of which scores of ≥ 5, ≥ 10, and ≥ 20 indicate mild, moderate, and severe depression, respectively [32]. We calculated median (IQR) for mastery scores, ranging from 7 to 28 with higher scores indicating greater levels of mastery [35]. We calculated mean differences in pre and during pandemic PHQ8 (mental health) and mastery scores and conducted paired t-tests testing the null hypothesis that the mean change in disability score was zero (p < 0.05). We examined associations between HDQ domain severity scores during the pandemic (6 scores), and frequency of living strategies use (51 items) using Spearman correlational analysis for non-normally distributed data. We computed 306 rank correlations and used a Bonferroni adjusted alpha ($\alpha_{adj} = 1.6 \times 10^{ - 4}$) to report the most significant correlations. We chose the severity scale of the HDQ as this scale possesses most measurement properties.

## Results

We recruited participants and administered the web-based survey between June 9 and August 12, 2020. Of 114 exercise study participants emailed to participate in the study, 60 (53%) clicked on the survey link (view rate), 59/60 (98%) consented to participate and initiated the questionnaires (participation rate), and 51/59 (86%) consented and completed the questionnaires (completion rate) [19]; and 12 additionally participated after word of mouth recruitment. Hence there was a total of 63 participants, 51 of which had HDQ, PHQ8 and Mastery scale data collected prior to the pandemic.

### Characteristics of participants and COVID-19 factors

The majority of participants were men (84%), White (65%), single (54%), and living alone (62%) with a median age 57 years (IQR: 49, 65 years). No participants reported testing positive for COVID-19.

#### Health care services/supports

Of the 61 (97%) participants who were receiving care from an HIV doctor or clinic, 71% received care remotely (telephone or online) during the COVID-19 pandemic (Table 1).

**Table 1:** Characteristics of participants, and COVID-19 pandemic health and support services, experiences and lifestyle changes (n = 63)

Characteristics	Number (%)
Median age (IQR)	57 years (49, 65)
Sex	
Male	53 (84%)
Female	10 (16%)
Gender	
Man	53 (84%)
Woman	9 (14%)
Non-binary	1 (2%)
Live alone	39 (62%)
Personal gross yearly income < $30,000 CAD	33 (52%)
Employment status	
Working for pay	18 (29%)
Retired	12 (19%)
On disability	18 (29%)
Most common concurrent conditions (≥ 30%)	
Mental health (e.g., depression, anxiety)	24 (38%)
Chronic pain (e.g., arthritis)	21 (33%)
Trouble sleeping (e.g., insomnia)	22 (35%)
Undetectable viral load (< 50 copies/mL)^a^	61 (97%)
Median number of years since HIV diagnosis (IQR)	22 (11, 32)
Overall self-reported health—very good or good	42 (67%)
Health status compared to previous year	
Better now than one year ago	19 (30%)
About the same as one year ago	25 (40%)
Worse now than one year ago	19 (30%)

COVID-19 pandemic—health care services/supports	
HIV care	
Remote (telephone/online)^b^ connect with HIV doctor or clinic during the pandemic	45 (71%)
Access to HIV medication impacted by pandemic	10 (16%)
Difficulties accessing healthcare	25 (40%)
Decreased health care services use during COVID	23 (37%)

COVID-19 pandemic—experiences and lifestyle changes	
Working during COVID-19	16 (25%)
Not working prior to pandemic (out of the 47 not working)	29 (62%)
Reduced wages or work hours	22 (35%)
Employment status changed since COVID-19 pandemic	20 (32%)
COVID-19 experiences	
Getting support from friends, family, partners, etc	33 (52%)
Fear of getting COVID-19	47 (75%)
Fear of giving COVID-19 to someone else	35 (56%)
Worrying about friends, family, partner, etc	54 (86%)
Frustration of boredom	50 (79%)
More anxiety	47 (75%)
More depression	34 (54%)
Changes to sleep patterns	37 (59%)
Alcohol use in past 30 days	34 (54%)
Cannabis use in past 30 days	24 (38%)
Lifestyle changes due to COVID-19 pandemic	
Practicing social distancing	60 (95%)
Isolating/quarantining from others	44 (70%)
Avoiding going to gym or exercise class	54 (86%)
Following media coverage on COVID	55 (87%)
Changing travel plans	44 (70%)
Stocking up on food and supplies	36 (57%)
Exercising less (frequency or intensity)	46 (73%)
Increase (frequency and amount) in substance use	14 (22%)

N = 63 participants’

^a^All participants were taking antiretroviral therapy medications

^b^Remote includes via telephone or video

#### Experiences and lifestyle changes

Sixteen (25%) participants reported working during the pandemic. Among those not working, most (29/47; 62%) were not working prior to the COVID-19 pandemic. Forty-five (71%) participants indicated that the COVID-19 pandemic impacted their day-to-day life either extremely (24%), very much (27%) or much (21%). During the pandemic, participants reported frustration of boredom (79%), increased anxiety (75%) and depression (54%), and changes to sleep patterns (59%). Thirty-three (52%) reported accessing emotional or social support from friends, family, partners, or counselors (Table 1).

### Disability during the pandemic

HDQ scores are presented in Table 2. Across all 63 participants, the highest median HDQ severity, presence and episodic (daily fluctuations of health challenges in the past week) scores were in the uncertainty, mental-emotional, and physical domains, respectively (Table 2).

**Table 2:** HIV disability questionnaire (HDQ) scores during the pandemic (n = 63 participants)

HDQ domain	Presence median (IQR)	Severity median (IQR)	Episodic median (IQR)
Physical symptoms	50 (25, 65)	16 (9, 25)	10 (0, 30)
Cognitive symptoms	66 (0, 100)	17 (0, 33)	0 (0, 33)
Mental-emotional health symptoms	73 (46, 91)	25 (14, 41)	9 (0, 36)
Uncertainty or worry about the future	64 (54, 86)	30 (16, 43)	0 (0, 14)
Difficulties with day-to-day activities	22 (0, 56)	11 (0, 25)	0 (0, 11)
Challenges to social inclusion	58 (25, 83)	23 (14, 48)	0 (0, 0)

HIV disability questionnaire (HDQ) score range; 0–100; higher scores indicate greater presence, severity and episodic nature of disability; n = 63 participants

### Change in disability (prior to versus during the pandemic)

**Fig. 1:**
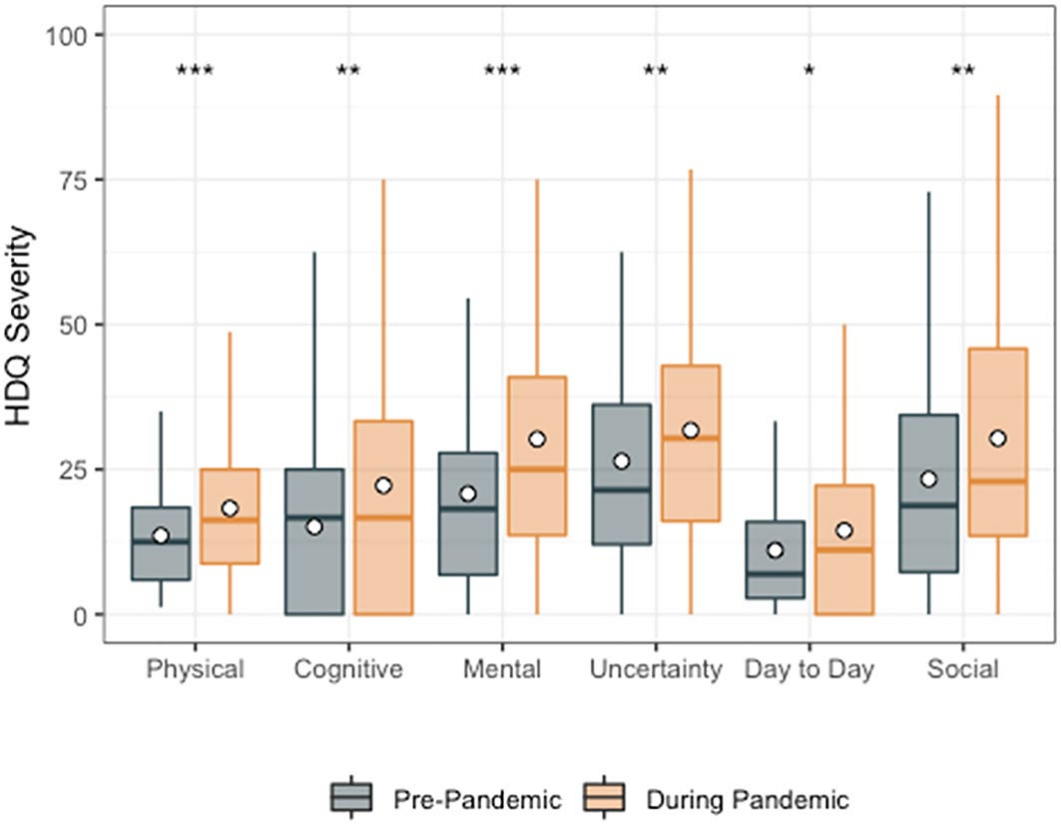
HDQ Severity mean domain scores prior to and during the pandemic (n = 51). Significance of paired t-tests shown above each domain as follows: ***p ≤ 0.001, **0.001 < p ≤ 0.01, *0.01 < p ≤ 0.05. n = 51 participants. Boxplots indicate median line and inter-quartile range (IQR), whiskers indicate 1.5 × IQR. Mean is shown as a circle

Among the 51 participants with pre-pandemic HDQ scores, mean HDQ severity scores were significantly higher (greater disability) during compared to prior to the pandemic, for all domains. The largest increase in disability severity was in the mental-emotional domain (mean HDQ change score: 11.4; sd: 15.3) (Fig. 1; Table 3).

**Table 3:** HDQ severity scores: pre versus during COVID-19 pandemic (n = 51)

HDQ domain	Spearman correlation coefficients	Pre-pandemic Mean (sd) HDQ severity scores	During pandemic Mean (sd) HDQ severity scores	Mean (sd) change in HDQ severity scores^a^	Paired *t*-test	p value
Physical	0.6	13.6 (9.3)	18.5 (12.0)	4.9 (9.6)	− 3.64	0.001***
Cognitive	0.6	15.1 (14.9)	21.9 (22.3)	6.8 (17.6)	− 2.76	0.008*
Mental-emotional	0.7	20.8 (18.0)	32.2 (21.3)	11.4 (15.3)	− 5.30	< 0.001***
Uncertainty	0.8	26.4 (20.3)	31.1 (22.0)	4.7 (11.5)	− 2.93	0.005**
Day-to-day activities	0.7	11.1 (11.9)	15.1 (18.5)	4.1 (11.9)	− 2.45	0.018*
Social inclusion	0.8	23.3 (18.7)	29.2 (23.0)	5.8 (14.2)	− 2.93	0.005**

HDQ severity scores range: 0–100; degrees of freedom: 50

^a^Mean change score (during—prior to pandemic for each participant)

^***^Indicates p ≤ 0.001

^**^Indicates 0.001 < p ≤ 0.01

^*^Indicates 0.01 < p ≤ 0.05

**Fig. 2:**
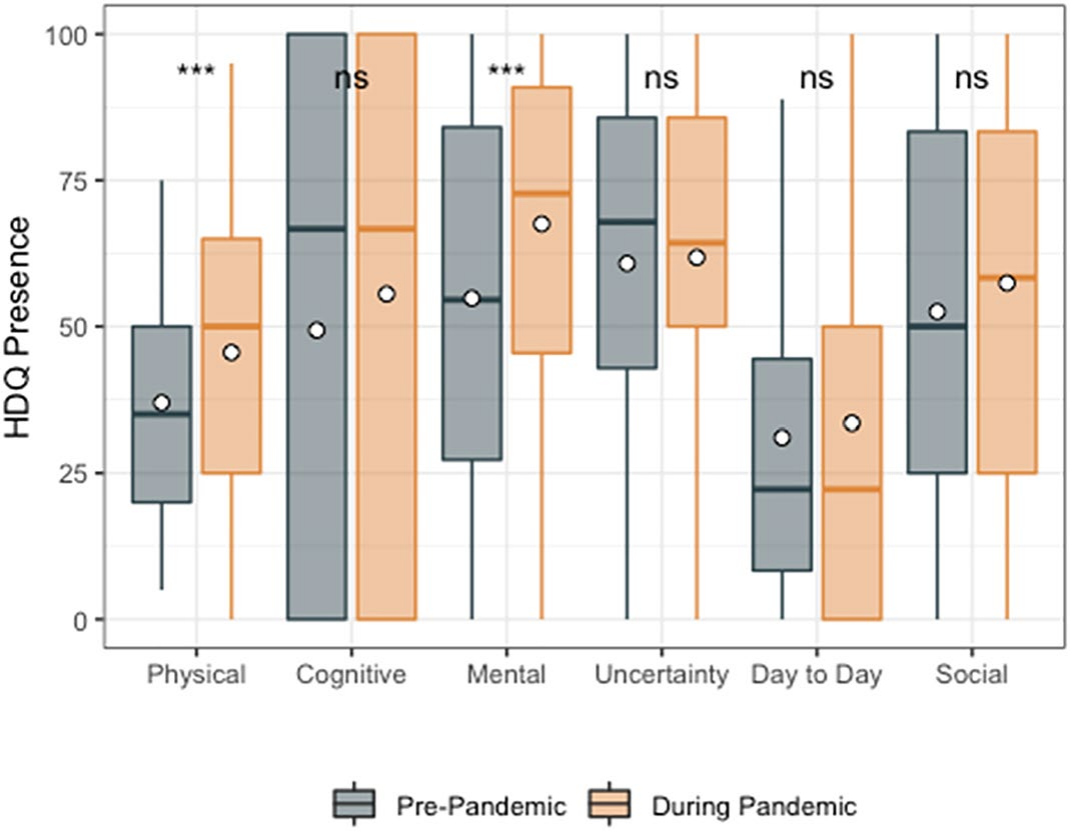
HDQ Presence mean domain scores prior to and during the pandemic (n = 51). Significance of paired *t*-tests is shown above each domain as follows: ***p ≤ 0.001, **0.001 < p ≤ 0.01, *0.01 < p ≤ 0.05 and ns indicates p > 0.05. n = 51 participants. Boxplots indicate median line and inter-quartile range (IQR), whiskers indicate 1.5 × IQR. Mean is shown as a circle

Among HDQ presence scores, only physical (mean HDQ change score: 10.3; sd: 20.7) and mental-emotional (mean HDQ change score: 17.7; sd: 26.2) were significantly higher (greater presence of disability) during compared to prior to the pandemic (Fig. 2; Table 4).

**Table 4:** HDQ presence scores: pre versus during COVID-19 pandemic (n = 51)

HDQ domain	Spearman correlation coefficients	Pre-pandemic mean (sd) HDQ presence scores	During pandemic mean (sd) HDQ presence scores	Mean (sd) change in HDQ presence scores	Paired *t*-test	p value
Physical	0.6	37.0 (20.9)	47.3 (24.8)	10.3 (20.7)	− 3.56	0.001***
Cognitive	0.6	49.3 (41.6)	56.2 (42.9)	6.9 (37.9)	− 1.29	0.202
Mental-emotional	0.6	54.8 (31.6)	72.5 (25.9)	17.7 (26.2)	− 4.84	< 0.001***
Uncertainty	0.8	60.8 (29.4)	61.8 (26.3)	1.0 (17.3)	− 0.41	0.687
Day-to-day activities	0.6	31.0 (28.0)	36.2 (34.1)	5.1 (26.0)	− 1.41	0.166
Social inclusion	0.8	52.5 (31.3)	56.7 (30.6)	4.2 (18.7)	− 1.59	0.118

HDQ presence scores range: 0–100; degrees of freedom: 50

*Indicates significant change score p < 0.05

^***^Indicates p < 0.001

^**^Indicates 0.001 < p ≤ 0.01

^*^Indicates 0.01 < p ≤ 0.05

**Fig. 3:**
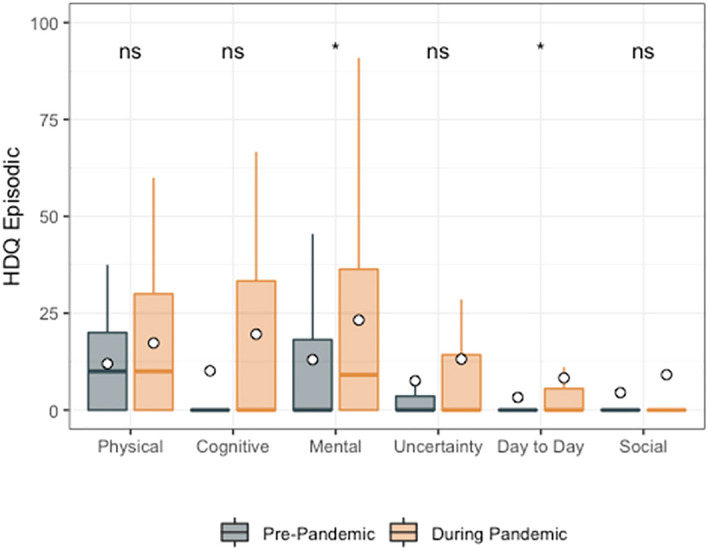
HDQ Episodic mean domain scores prior to and during the pandemic (n = 51). Significance of paired *t*-tests is shown above each domain as follows: ***p < 0.001, **0.001 < p ≤ 0.01, * 0.01 < p ≤ 0.05 and ns indicates p > 0.05. n = 51 participants. Boxplots indicate median line and inter-quartile range (IQR), whiskers indicate 1.5 × IQR. Mean is shown as a circle

Among HDQ episodic scores, only mental-emotional (mean HDQ episodic change score: 9.3; sd: 30.6) and difficulties carrying out day-to-day activities (mean HDQ episodic change score: 5.2; sd: 16.2) were higher during compared to prior to the pandemic (Fig. 3; Table 5).

**Table 5:** HDQ episodic scores: pre versus during COVID-19 pandemic (n = 51)

HDQ domain	Spearman correlation coefficients	Pre-pandemic mean (sd) HDQ episodic scores	During pandemic mean (sd) HDQ episodic scores	Mean (sd) change in HDQ episodic scores	Paired *t*-test	p value
Physical	0.5	12.0 (14.3)	16.7 (18.9)	4.7 (18.9)	− 1.78	0.082
Cognitive	0.5	10.1 (23.1)	17.6 (33.6)	7.5 (29.5)	− 1.82	0.075
Mental-emotional	0.4	13.0 (22.6)	22.3 (30.5)	9.3 (30.6)	− 2.17	0.035*
Uncertainty	0.4	7.6 (18.4)	10.9 (19.7)	3.4 (16.0)	− 1.50	0.140
Day-to-day activities	0.4	3.3 (8.8)	8.5 (18.4)	5.2 (16.2)	− 2.30	0.026*
Social inclusion	0.4	4.5 (14.9)	5.6 (17.7)	1.1 (13.7)	− 0.55	0.582

HDQ episodic scores range: 0–100; degrees of freedom: 50

^***^Indicates p < 0.001

^**^Indicates 0.001 < p ≤ 0.01

^*^Indicates 0.01 < p ≤ 0.05

#### Secondary measures

Mental health (PHQ8) scores increased from 6.2 (pre-pandemic) to 8.0 (during the pandemic), classified as ‘mild depression’ at both time points (change score: 1.9 points; p = 0.01) (Additional File 1). Across all depression categories of the PHQ8, 20 of the 51 participants (39%) reported increased depression during the pandemic, 6 (12%) reported less depression, and 25 (49%) remained stable (Additional File 2). There was no change in mastery scores (Additional File 1).

### Living strategies

The majority of participants (> 60%) reported engaging in positive living strategies a ‘few times a week’ or ‘everyday’ pertaining to maintaining a sense of control (represented in 14 different strategies in areas of maintaining a healthy lifestyle, maintaining health as focus and purpose in life, maintaining life balance, planning for and anticipating the future, and paying attention to viral load to remain on top of health) and adopting positive attitudes and beliefs (represented by five different strategies related to positive outlook) (Additional File 3). Nine strategies (18%) changed since the onset of the pandemic by the majority of participants (> 50%), most of which pertained to social interaction (decreasing time spent with friends, colleagues or seeking company with others, increasing isolation, and increasing time interacting with others on the internet). Forty-eight percent of participants reported increasing their use of interacting with others on the internet, and 27% engaged in this strategy ‘a few times a week’ or ‘everyday’ (Additional File 3).

### Associations between disability and self-care living strategies during the COVID-19 pandemic

All domains of the HDQ except the physical domain were significantly associated (Spearman rho |≥ 0.46|) with frequency of use of eight living strategies. Maintaining a sense of control (focusing on things such as work, friends and activities (Spearman rho | 0.47|), maintaining a good balance of activity in life (Spearman rho | 0.51|), and trying to stick to daily structure or routine (Spearman rho | 0.46|), were associated with lower HDQ (improved) severity scores for mental-emotional health challenges. Living strategies pertaining to attitudes and beliefs (considering self to be healthy (Spearman rho | 0.50–0.60|), accepting and valuing who I am (Spearman rho | 0.47–0.52|), positive outlook on life (Spearman rho | 0.46–0.54|), choosing to believe one can overcome any challenges (Spearman rho | 0.47–0.53|), were associated with lower (improved) HDQ severity scores for challenges with mental-emotional and cognitive health, uncertainty, day-to-day activities, and social inclusion, and feeling hopeless (Spearman rho | 0.46–0.62|) was associated with greater (worse) HDQ severity scores for challenges with mental-emotional and cognitive health, and social inclusion (Additional File 4).

## Discussion

Using a previously validated disability questionnaire with adults with HIV administered prior to the pandemic, we found that self-reported disability severity increased across all dimensions during the COVID-19 pandemic among adults living with HIV, with the largest increase found in the presence, severity, and episodic components of mental-emotional health. This finding was supported by the significant increase in mean PHQ8 scores and 39% reporting a higher category of depression during the pandemic compared to prior (Additional files 1 and 2). Similarly, the majority of participants reported ‘more anxiety’, ‘more depression’, or ‘frustration of boredom’ during the pandemic (Table 1).

Uncertainty followed by mental-emotional health challenges were the most severe dimensions of disability experienced during the pandemic (Table 2). These dimensions of disability are closely related as uncertainty is a strong predictor of mental-emotional health challenges for adults with HIV [37–39]. The increase in uncertainty scores during the pandemic were supported by participants who reported fear of getting COVID-19 (75%), worrying about friends, family and partner (86%) (Table 1), suggesting factors that may be contributing to disability dimensions of uncertainty and mental-emotional health challenges in the HDQ scores. The concept of uncertainty in COVID-19 has been explored specifically as it pertains to diagnostic uncertainty and unknown severity, duration, and long-term impact of symptoms associated with COVID-19 [40–42]. The impact of uncertainty associated with an ongoing pandemic, uncertainty of acquiring COVID-19 and subsequently Long COVID, and ongoing safety measures and policy among individuals health and well-being during the pandemic is unclear. Uncertainty increased in severity but not presence, suggesting that existing uncertainty was exacerbated by the pandemic. Adults with HIV may have prior experiences living with uncertainty from the earlier days of the HIV epidemic [38, 39, 43]. However, the extent to which these prior experiences may exacerbate or equip an individual with strategies to deal with uncertainty during a new (COVID-19) pandemic is unknown and an area of future research.

In addition to the greater risk and poorer outcomes of COVID-19 that may occur among people with HIV [44–47], the pandemic can further exacerbate stress and isolation, unemployment, food insecurity, access to care, and difficulty managing underlying comorbidities [9, 48]. These factors may amplify disability, as reflected by the increase of HDQ severity scores across all dimensions in the sample. Evidence is emerging on the impact of HIV and COVID-19 as co-pandemics, including the impact of co-infection on health services [14]. Further qualitative inquiry may help to explore the nature and impact of the pandemic on health outcomes for adults with HIV.

The greatest change in disability was in the mental-emotional health domain, where participants experienced the largest increases in presence, severity and episodic scores of disability compared with pre-pandemic scores. Physical distancing measures and public health restrictions during the COVID-19 pandemic have been linked to loneliness and social isolation among adults with HIV [49], which may have exacerbated mental and emotional health challenges and challenges to social inclusion. Most participants were practising social distancing, reflected in the most common changes in living strategies related to social interaction. While 48% of participants reported increasing their use of interacting with others on the internet, only 27% frequently engaged in this strategy (Additional file 3). Given the majority of participants lived alone, this may further highlight challenges to social inclusion and mental-emotional health experienced during the pandemic. COVID-19 restrictions may additionally pose difficulties for attending medical appointments, or accessing medications, subsequently affecting the health of adults living with HIV [9, 10, 13]. While most participants remained engaged in care by accessing their HIV doctor remotely (71%), some reported difficulties accessing (40%) and using (37%) health care services (Table 1). Of note, the pandemic appeared to have less of an impact on employment, which is likely a reflection of almost half of the participants (46%) who were not working prior to the pandemic.

The majority of participants reported sustained use of positive living strategies during the pandemic with the exception of positive social interaction strategies and negative strategies (e.g., isolating self) that increased during the pandemic (Additional file 3). Another study similarly reported that higher levels of social support and resilient coping were associated with lower depressive symptoms among adults with HIV in the context of the COVID-19 pandemic [50]. The increase in social isolation (spending less time with support networks, less time going out or spending time with others, and tending to isolate self more), a likely consequence of the COVID-19 restrictions, aligns with the increase in disability in dimensions of social inclusion and mental-emotional health as measured by the HDQ. This highlights the role for remote social support and services that can promote social interaction and support among community during the pandemic.

To our knowledge this is the first study to explore the nature, extent and impact of disability experienced by adults living with HIV prior to and during the COVID-19 pandemic, and to specifically explore the impact of the COVID-19 pandemic on health outcomes and living strategies use for people with chronic illness. This work provides insights to the health challenges experienced by adults with HIV who may be living with the added complexity of concurrent health conditions [51–53], and specifically the common mental health conditions such as anxiety and depression. Opportunities exist for health providers to apply the lessons learned in HIV rehabilitation to the context of disability experienced during the COVID-19 pandemic, such as anticipating and preparing for the impact of disability, acknowledging the episodic nature in which disability may be experienced, recognizing the impact of stigma and health inequities, understanding uncertainty in COVID-19 related disability, and implementing disability and rehabilitation-focused responses that include people affected by the COVID-19 pandemic that may help health providers better address the needs of people living with HIV during the COVID-19 pandemic [43, 54, 55].

We administered study questionnaires in-between a first (Spring 2020) and second (Fall 2020) wave of the COVID-19 pandemic in Toronto, Ontario. With COVID-19 restrictions continuing with third and fourth waves in Spring and Summer 2021, the sustained and potential cumulative impact of COVID-19 on disability dimensions are unclear. The clinical importance of HDQ change scores should be interpreted with caution. Pre-pandemic measures were collected from 2016 to 2018, hence it is unknown to what extent changes may be attributed to other contextual factors, such aging over time.

While a strength of our study was building on data collected pre-pandemic (HDQ, PHQ8, mastery) with an existing cohort of adults living with HIV who participated in an earlier exercise intervention study, our study is not without limitations. We assessed disability and living strategies use cross-sectionally during the pandemic, as distinct constructs and cannot infer causation between the pandemic and disability and living strategies use. Furthermore, the study did not include an HIV-negative control group, hence it is unknown whether changes in disability among adults living with HIV differ from the broader population. Due to our small sample size, our analysis was exploratory. Furthermore, the clinical importance of HDQ change scores, particularly given the episodic nature of disability in the context of the COVID-19 pandemic is unclear. Nevertheless, our inclusion of supplemental measures of mental health (PHQ8) support the direction of change in disability this study. Given the majority of the sample were from a previous study examining the impact of community-based exercise among adults living with HIV, primarily including White men living in an urban setting, results may not be representative of the larger HIV population. Men and women can experience disability differently pertaining to hospitalization, parental roles, fatigue, mental health, and social inclusion [56–58]. Future work should examine the influence of gender on the disability experiences among adults with HIV in the context of the pandemic.

## Conclusions

Participants with HIV reported high levels of uncertainty and mental health challenges during the pandemic. All six dimensions of disability increased during the pandemic, the largest increase was the mental-emotional health domain. Strategies involving maintaining a sense of control, and positive attitudes and beliefs were associated with lower disability during the pandemic. Results help to provide an understanding of disability and self-care living strategy use among adults living with HIV during the COVID-19 pandemic.

## Supplementary Information

**Additional file 1.** PHQ8 (mental health) and mastery scores—pre and during the COVID-19 pandemic.

**Additional file 2.** Participant change in PHQ8 classification category of depression pre and during the pandemic (n = 51 participants).

**Additional file 3.** Living strategies use and change of use since the COVID-19 pandemic (n = 63).

**Additional file 4.** Associations between disability severity and self-care living strategies use during the pandemic (n = 63 participants).

## Data Availability

The data used and/or analysed during the current study are available from the corresponding author on reasonable request. The data supporting the conclusions of this article are included within the article and its additional files.

## Abbreviations

HIV: Human Immunodeficiency Virus
HDQ: HIV disability questionnaire
IQR: Interquartile range
PHQ8: Patient health questionnaire
SD: Standard deviation
