# Supplementary material for: Disability and self-care living strategies among adults living with HIV during the COVID-19 pandemic
Source: AIDS Res Ther. 2021 Nov 19;18:87. doi: 10.1186/s12981-021-00413-4 (PMC8604195; doi:10.1186/s12981-021-00413-4)
Supplement: Supplementary file 3 — Additional file 3. Living strategies use and change of use since the COVID-19 pandemic (n = 63). [file 12981_2021_413_MOESM3_ESM.pdf]

Additional File 3 – Living Strategies Use and Change of Use since the COVID-19 Pandemic (n=63)

LIVING STRATEGY ITEM	Strategy conceptualized as having Positive (+) or Negative (-) influence on health	Scale A: In the last month, how often have you used the following strategies?				Scale B: Has your use of this strategy changed since the onset of the COVID-19 pandemic?		
		None of the time (e,g. not at all)	A little of the time (e.g. few times a month)	Some of the time (e.g. few times a week)	All of the time (e.g. everyday)	Yes, increased / more frequent	Yes, decreased / less frequent	No change
**Maintaining sense of control (26 items)**								
**Lifestyle**								
1) I make sure I get enough sleep.	+	3 (5%)	9 (14%)	15 (24%)	36 (57%)	22 (35%)	8 (13%)	33 (52%)
2) I take a break or nap when I need to.	+	6 (10%)	14 (22%)	26 (41%)	17 (27%)	30 (48%)	8 (13%)	25 (40%)
3) I make sure I eat healthy.	+	2 (3%)	11 (18%)	21 (33%)	29 (46%)	17 (27%)	11 (18%)	35 (56%)
4) I brush my teeth and practice good dental care.	+	1 (2%)	7 (11%)	15 (24%)	40 (64%)	13 (21%)	7 (11%)	43 (68%)
5) I exercise.	+	10 (16%)	20 (32%)	21 (33%)	12 (19%)	14 (22%)	29 (46%)	20 (32%)
6) I take medications as discussed with my doctor.	+	2 (3%)	1 (2%)	7 (11%)	53 (84%)	5 (8%)	3 (5%)	55 (87%)
7) I try and avoid people or things that stress me out.	+	2 (3%)	7 (11%)	29 (46%)	25 (40%)	25 (40%)	5 (8%)	33 (52%)
8) I smoke cigarettes.	-	53 (84%)	1 (2%)	0 (0%)	9 (14%)	7 (11%)	2 (3%)	54 (86%)
**Maintain Focus / Establishing Purpose**								
9) I focus on a hobby	+	10 (16%)	25 (40%)	16 (25%)	12 (19%)	17 (27%)	11 (18%)	35 (56%)
10) I focus on maintaining my health.	+	2 (3%)	9 (14%)	21 (33%)	31 (49%)	19 (30%)	8 (13%)	36 (57%)
11) I focus on things such as work, friends and activities.	+	7 (11%)	14 (22%)	25 (40%)	17 (27%)	11 (18%)	15 (24%)	37 (59%)
12) I make HIV the main focus of my life.	+/-	34 (54%)	17 (27%)	8 (13%)	4 (6%)	7 (11%)	6 (10%)	50 (80%)
13) I make maintaining my health the main focus of my life.	+/-	5 (8%)	18 (29%)	15 (24%)	25 (40%)	12 (19%)	11 (18%)	40 (64%)
14) I am involved in volunteerism, activism or advocacy related to HIV.	+	27 (43%)	18 (29%)	9 (14%)	9 (14%)	5 (8%)	16 (25%)	42 (67%)
15) I engage in activities of artistic expression (such as drawing, painting, dancing, singing, chanting, creative writing, etc.).	+	31 (49%)	13 (21%)	13 (21%)	6 (10%)	8 (13%)	12 (19%)	43 (68%)
16) I try to manage my finances.	+	4 (6%)	10 (16%)	10 (16%)	39 (62%)	7 (11%)	3 (5%)	53 (84%)

**Maintaining Life Balance**								
17) I maintain a good balance of activity in my life.	+	8 (13%)	16 (25%)	22 (35%)	17 (27%)	13 (21%)	18 (29%)	32 (51%)
18) I try and stick to daily structure or routine.	+	5 (8%)	22 (35%)	16 (25%)	20 (32%)	11 (18%)	23 (37%)	29 (46%)
19) I prioritize and try not to overdo it when it comes to daily activities.	+	6 (10%)	11 (18%)	21 (33%)	25 (40%)	16 (25%)	9 (14%)	38 (60%)
20) I tend to overdo it by working or volunteering long hours.	-	44 (70%)	12 (19%)	4 (6%)	3 (5%)	8 (13%)	13 (21%)	42 (67%)
**Planning for and Anticipating the Future**								
21) I plan for and anticipate changes in my health.	+	18 (29%)	20 (32%)	17 (27%)	8 (13%)	12 (19%)	6 (10%)	45 (71%)
22) I plan ahead at work or in my daily routine for possible fluctuations in my health.	+	28 (44%)	16 (25%)	13 (21%)	6 (10%)	9 (14%)	6 (10%)	48 (76%)
23) I make to-do lists to help keep on track with my daily routine.	+	18 (29%)	17 (27%)	13 (21%)	15 (24%)	10 (16%)	15 (24%)	38 (60%)
24) I learn what I can about COVID-19 so that I am informed.	+	5 (8%)	4 (6%)	20 (32%)	34 (54%)	30 (48%)	4 (6%)	29 (46%)
**Paying attention to the numbers**								
25) I pay attention to "the numbers" (for example, my viral load and CD4 counts) as a way to keep on top of my health.	+	8 (13%)	15 (24%)	8 (13%)	32 (51%)	7 (11%)	7 (11%)	49 (78%)
26) I use websites or applications (apps) to track my details (counts, medications, other conditions).	+	38 (60%)	9 (14%)	8 (13%)	8 (13%)	4 (6%)	6 (10%)	53 (84%)
**Attitudes and Beliefs (8 items)**								
**Positive Outlook**								
27) I consider myself healthy.	+	7 (11%)	12 (19%)	21 (33%)	23 (37%)	6 (10%)	12 (19%)	45 (71%)
28) I accept and value who I am – the good and the bad.	+	1 (2%)	16 (25%)	18 (29%)	28 (44%)	14 (22%)	10 (16%)	39 (62%)
29) I have a positive outlook on life and use hope and optimism.	+	2 (3%)	16 (25%)	19 (30%)	26 (41%)	10 (16%)	12 (19%)	41 (65%)
30) I accept that my health can fluctuate with good days and bad days.	+	2 (3%)	13 (21%)	13 (21%)	35 (56%)	9 (14%)	8 (13%)	46 (73%)
31) I choose to believe I can survive and overcome any challenges.	+	2 (3%)	13 (21%)	25 (40%)	23 (37%)	11 (18%)	12 (19%)	40 (64%)
32) I get upset if I don't get to everything I set out to do in a day.	-	20 (32%)	20 (32%)	19 (30%)	4 (6%)	11 (18%)	7 (11%)	45 (71%)
33) I feel hopeless.	-	29 (46%)	22 (35%)	7 (11%)	5 (8%)	15 (24%)	8 (13%)	40 (64%)
**Faith and Spirituality**								
34) I draw on faith and spirituality.	+	19 (30%)	17 (27%)	15 (24%)	12 (19%)	10 (16%)	9 (14%)	44 (70%)

**Blocking the pandemic out of the mind (7 items)**								
35) I ignore the COVID-19 pandemic.	-	43 (68%)	10 (16%)	7 (11%)	3 (5%)	9 (14%)	5 (8%)	49 (78%)
36) I ignore my numbers (viral load and CD4 count).	-	46 (73%)	7 (11%)	3 (5%)	7 (11%)	4 (6%)	3 (5%)	56 (89%)
37) I drink alcohol as an escape to forget the COVID-19 pandemic.	-	48 (76%)	8 (13%)	4 (6%)	3 (5%)	12 (19%)	3 (5%)	48 (76%)
38) I try to forget the COVID-19 pandemic.	+/-	34 (54%)	17 (27%)	8 (13%)	4 (6%)	9 (9%)	6 (10%)	48 (76%)
39) I use drugs recreationally to forget the COVID-19 pandemic.	+/-	50 (79%)	9 (14%)	3 (5%)	1 (2%)	9 (14%)	1 (2%)	53 (84%)
40) I use sex as a way to forget the COVID-19 pandemic.	+/-	52 (83%)	8 (13%)	1 (2%)	2 (3%)	8 (13%)	5 (8%)	50 (79%)
41) I use online shopping to forget the COVID-19 pandemic.	+/-	44 (70%)	14 (22%)	3 (5%)	2 (3%)	13 (21%)	3 (5%)	47 (75%)

**SOCIAL INERATION LIVING STRATEGY ITEMS**	**Strategy conceptualized as having Positive (+) or Negative (-) influence on health**	**Scale A: In the last month, how often have you used the following strategies?**						**Scale B: Has your use of this strategy changed since the onset of the COVID-19 pandemic?**			
		**None of the time (e.g. not at all)**	**A little of the time (e.g. few times a month)**	**Some of the time (e.g. weekly)**	**Most of the time (e.g. few times a week)**	**All of the time (e.g. everyday)**	**Not applicable**	**Yes, increased / more frequent**	**Yes, decreased / less frequent**	**No change**	**Not applicable**
**Social Interaction (10 items)**											
42) I spend time with my friends, partner, extended chosen or biological family, or pets.	+	9 (14%)	18 29%)	14 (22%)	6 (10%)	14 (22%)	2 (3%)	15 (24%)	18 (29%)	25 (40%)	5 (8%)
43) I spend time with work (or volunteer) colleagues.	+	35 (56%)	16 (25%)	3 (5%)	3 (5%)	0 (0%)	6 (10%)	3 (5%)	20 (32%)	30 (48%)	10 (16%)
44) I spend time with people I know through religion or faith.	+	44 (70%)	5 (8%)	6 (10%)	0 (0%)	0 (0%)	8 (13%)	3 (5%)	5 (8%)	38 (60%)	17 (27%)
45) I spend time at a community-based or AIDS service organization, either to volunteer or access their services.	+	36 (57%)	16 (25%)	5 (8%)	1 (2%)	2 (3%)	3 (5%)	6 (10%)	14 (22%)	35 (56%)	8 (13%)

46) I seek the company of others living with HIV (outside of organizations).	+	33 (52%)	15 (24%)	11 (18%)	2 (3%)	0 (0%)	2 (3%)	4 (6%)	12 (19%)	38 (60%)	9 (14%)
47) I spend time interacting with others on the internet (e.g. Zoom, FaceTime, Facebook, Twitter, Instagram etc.)	+	11 (18%)	12 (19%)	22 (35%)	9 (14%)	8 (13%)	1 (2%)	30 (48%)	5 (8%)	24 (38%)	4 (6%)
48) I seek the company of others by attending dinner parties or going out to restaurants.	+	50 (79%)	8 (13%)	0 (0%)	0 (0%)	0 (0%)	5 (8%)	8 (13%)	19 (30%)	29 (46%)	7 (11%)
49) I access a support group (either online, by telephone or in person).	+	43 (68%)	9 (14%)	7 (11%)	1 (2%)	1 (2%)	2 (3%)	3 (5%)	8 (13%)	43 (68%)	9 (14%)
50) I isolate myself from others.	-	11 (18%)	12 (19%)	10 (16%)	15 (24%)	12 (19%)	3 (5%)	21 (33%)	4 (6%)	30 (48%)	8 (13%)
51) I try to reduce or eliminate relationships or activities that I believe are harmful to me.	+	7 (11%)	3 (5%)	10 (16%)	14 (22%)	23 (37%)	6 (10%)	18 (29%)	1 (2%)	36 (57%)	8 (13%)

LEGEND: Yellow Highlights: indicates strategies where most (>60%) participants tended to engage most of the time (few times a week or all the time (everyday) in a "healthy" living strategy (n=19 different strategies); **Green Highlights**: indicates strategies where there was a change in strategy use among >50% of the sample during pandemic (n=9 different strategies).
